# Enhanced Fault Type Detection in Covered Conductors Using a Stacked Ensemble and Novel Algorithm Combination

**DOI:** 10.3390/s23208353

**Published:** 2023-10-10

**Authors:** Ondřej Kabot, Lukáš Klein, Lukáš Prokop, Wojciech Walendziuk

**Affiliations:** 1 ENET Centre—CEET, VSB—Technical University of Ostrava, 708 00 Ostrava, Czech Republic; lukas.klein@vsb.cz (L.K.); lukas.prokop@vsb.cz (L.P.); 2 Department of Computer Science, VSB—Technical University of Ostrava, 708 00 Ostrava, Czech Republic; 3 Faculty of Electrical Engineering, Bialystok University of Technology, 15-351 Bialystok, Poland; w.walendziuk@pb.edu.pl

**Keywords:** partial discharge, covered conductors, radio antenna, frequency domain analysis, fault diagnosis

## Abstract

This study introduces an innovative approach to enhance fault detection in XLPE-covered conductors used for power distribution systems. These covered conductors are widely utilized in forested areas (natural parks) to decrease the buffer zone and increase the reliability of the distribution network. Recognizing the imperative need for precise fault detection in this context, this research employs an antenna-based method to detect a particular type of fault. The present research contains the classification of fault type detection, which was previously accomplished using a very expensive and challenging-to-install galvanic contact method, and only to a limited extent, which did not provide information about the fault type. Additionally, differentiating between types of faults in the contact method is much easier because information for each phase is available. The proposed method uses antennas and a classifier to effectively differentiate between fault types, ranging from single-phase to three-phase faults, as well as among different types of faults. This has never been done before. To bolster the accuracy, a stacking ensemble method involving the logistic regression is implemented. This approach not only advances precise fault detection but also encourages the broader adoption of covered conductors. This promises benefits such as a reduced buffer zone, improved distribution network reliability, and positive environmental outcomes through accident prevention and safe covered conductor utilization. Additionally, it is suggested that the fault type detection could lead to a decrease in false positives.

## 1. Introduction

Partial discharges (PD) are a prevalent phenomenon that can occur in distribution lines equipped with covered conductors (CC) [1]. These discharges can result in insulation failure, leading to a range of problems such as equipment damage and safety hazards [2]. PDs manifest as low-energy, high-frequency pulses capable of eroding insulation and causing its breakdown [3]. The causes behind PD activity are diverse, encompassing factors like aging, contamination, mechanical stress, improper cable joining, and contact with surrounding vegetation [4].

### 1.1. Covered Conductors and Their Challenges

Covered conductors offer an alternative to bare conductors by integrating insulated cores, usually employing cross-linked polyethylene (XLPE) insulation. The installation of these types of conductors is primarily motivated by the requirement for reduced spacing between individual phases. This leads to narrower buffer zones, resulting in environmental conservation [5] as well as financial savings [6]. These covered conductors are usually used in natural parks and heavily forested areas in order to reduce maintenance costs and the size of the buffer zone.

**Figure 1:**
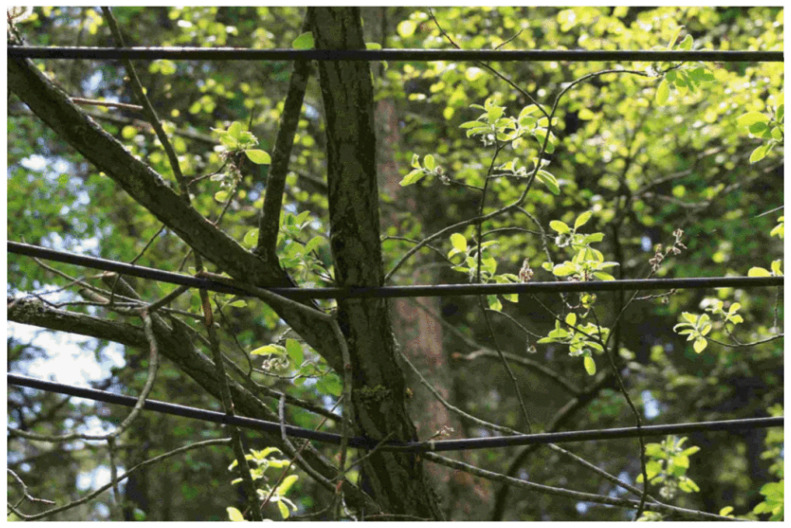
Example of high-impedance fault caused by tree branches [3].

Another significant factor is the potential occurrence of high-impedance faults and subsequent PD activity when adjacent vegetation comes into contact with these covered conductors. In contrast, if such an incident were to happen on a line equipped with bare conductors, it would lead to a ground fault and, in the best-case scenario, a power outage. Although the covered conductor would prevent the immediate ground fault, prolonged contact (such as from a fallen tree on the power line as can be seen in Figure 1), would result in PDs that could eventually lead to insulation breakdown of the covered conductor [7].

However, challenges arise when attempting to detect these conditions while covered conductors are in contact with vegetation. This difficulty stems from a relatively low amplitude of the generated pulses, making their detection a complex task.

Various methods (radiometric [8], galvanic contact [9], using SDR [10], acoustic [11], and optical using a UVC-sensitive camera [12], high-frequency current transformer [13]) for identifying these pulses in covered conductors exist, and the market offers commercially available solutions for this purpose. However, some of them are not usable for distribution line monitoring (acoustic or optical); these methods are most useful in cases of direct maintenance work when the line is checked by personnel. It is worth noting that many of these solutions are notably expensive and often cannot accurately classify the type of fault. Moreover, it is best to have online detection on the site itself to reduce the maintenance costs [13,14,15].

PD signals are made up of signals of various frequency components, including frequency components in the GHz region. When PD signals travel for a few wavelengths, the cable starts to act like an antenna and begins to show traveling wave radiation patterns [16,17]. In addition, these patterns are specific and can also be used to localize fault [18].

### 1.2. Motivation for the Study

Although the detection and analysis of PDs are areas undergoing constant development and scientific research, there are still numerous opportunities for improvement [19,20]. The low energy of the pulses makes them challenging to detect, as there is a high likelihood that some of the pulses will be obscured by the surrounding noise [21]. This challenge is exacerbated by the increasing number of wireless devices and the growing demand for connectivity. Another issue pertains to the cost of most commercially available solutions. Another significant issue is the fact that these lines are mostly used in forested and hilly areas, which makes them difficult to access and maintain. The installation of any new device can pose a challenge and often requires a power line shutdown. Wireless systems can be designed in such a way that there is no need for a power line shutdown.

Additionally, many of these solutions lack the capability for fault type classification, a feature crucial for determining the severity of a fault and scheduling maintenance to rectify its source. The classification of fault types also remains an insufficiently explored area, with only a limited number of articles addressing the problem. Even if article [22] attempts fault type detection, it does not detect faults with a ground connection and relies on the relatively expensive galvanic contact method [15], which captures signals from all three phases, and makes it much easier to detect faults in different phases. However, this issue is not discussed in the literature There are a few tangentially related studies focusing on cable joints [23], workmanship defects [24], and bare conductors using simulated data [25]. None of them use an antenna-based method [26], which offers many benefits for detecting the type of fault in covered conductors in power lines. This is of interest in this study, as it involves unique types of faults. What is more, we have two large datasets (one has already been published [27], and the second one is planned to be published, but is partially present in Kaggle competition (https://www.kaggle.com/c/vsb-power-line-fault-detection, accessed on 4 October 2023); however, it was used in some previous works [22]) for the detection of partial discharges in distribution transmission lines. Both of them lack annotation for fault type and only have labels for fault occurrence.

Therefore, we want to verify whether it is possible, using only an antenna-based radiometric method, to distinguish between types of faults and thereby increase the reliability and applicability of the method. The aim of this research is to distinguish between various types of faults that can occur on isolated distribution lines equipped with CCs operating at a voltage level of 22 kV. The considered fault types include line-to-ground, two lines-to-ground, three lines-to-ground, line-to-line, and interconnected faults involving all lines. The knowledge about the fault type can be valuable for distribution line operators, enabling them to assess the severity of the fault and plan maintenance for the compromised line. Furthermore, it can help to identify the root cause of the fault. For instance, a fault caused by a tree leaning onto the distribution line would likely exhibit the characteristics of a high-impedance ground fault. However, a plausible explanation would involve a foreign object interconnecting individual lines if the fault occurred only between lines.

The ultimate goal of our continuous research is to develop a cost-effective solution that is easy to install and provides comprehensive information about faults on the distribution line. Such a solution would enhance the reliability and safety of the distribution network, consequently reducing the downtime required for maintenance due to insulation failures and lower operating costs.

### 1.3. Research Objectives

In this section, we outline our primary research objectives, which center around the utilization of radiometric antenna detection. Our aim is to assess the feasibility of detecting and classifying various types of faults using this approach. The specific objectives are as follows:

1. Determine the feasibility of detecting fault events, particularly those of low energy, through radiometric antenna detection, as this has not been achieved before with this method.
2. Explore the capabilities of the radiometric antenna–spectrometer system in classifying different types of faults, including line-to-ground, two lines-to-ground, three lines-to-ground, line-to-line, and interconnected faults.
3. Confirm that differentiating between types of faults can improve the reliability of fault detection itself.

By addressing these research objectives, we aim to contribute to the advancement of fault type detection and classification technologies for distribution lines with CC, with a specific focus on utilizing radiometric antenna detection and the classification of different types of faults. This would enable the cheaper detection and wider usage of distribution lines with CC. This research will provide a valuable insight into the potential of this approach and its practical implementation for enhancing distribution network operations and reducing downtime associated with fault-related maintenance.

## 2. Materials and Methods

In this section, we will describe the approach we employed to conduct our experiments and outline the resources we utilized to fulfill our research objectives.

### 2.1. Description of Experimental Setup

**Figure 2:**
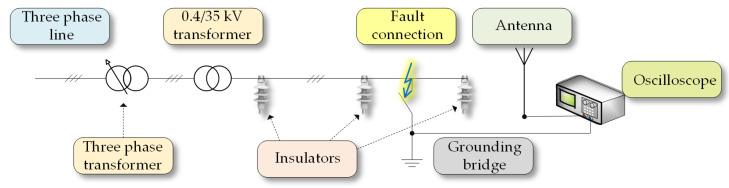
One-pole schematic.

For the purpose of this measurement, a setup that replicated the real-life conditions was utilized. The only difference was the varying distance of the individual lines from the ground and the means of fault was a stainless steel rod, which as a measurement with tree branches would be hard to replicate. The entire measurement setup comprised an adjustable 3-phase auto-transformer connected to the low-voltage side of a 0.4/35 kV dry insulated transformer. The high-voltage output of this transformer was subsequently connected to the individual CC lines, as illustrated in Figure 2. The cross-section of the CC used in this experiment was 35 mm${}^{2}$ and its core consisted of seven individual aluminum wires. The length of individual lines was approximately 8.5 m.

Each line was affixed to a set of ceramic, pin-type insulators that are commonly used in distribution networks with a nominal voltage of 22 kV. At both ends of the line, stainless steel spheres were attached to ensure a geometrically graded electrical field, thereby preventing the occurrence of PDs in unwanted areas. A similar approach was adopted for the high-voltage terminals of the 35 kV transformer. To verify the correct installation, the assessment was conducted using a UVC-sensitive camera.

To facilitate the detection of PDs, an antenna was utilized. It was positioned perpendicular to the CC lines, located a meter’s distance away from them. This antenna was subsequently linked to an oscilloscope Siglent SDS-5034X (manufacturer Siglent, Helmond, The Netherlands), serving as the data acquisition platform.

**Figure 3:**
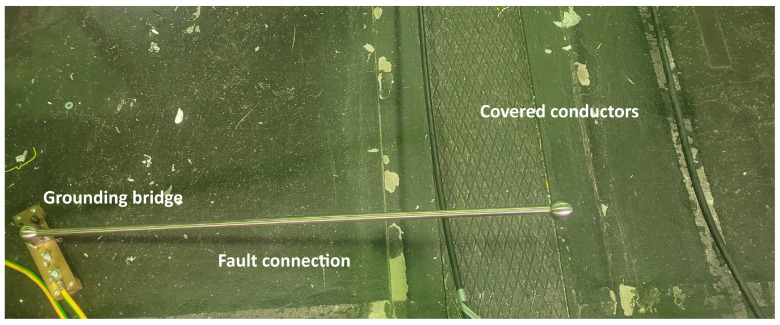
High-impedance fault of the line to ground.

To simulate a fault, a stainless steel tube of ample length was employed to establish contact between phases that were involved in the fault type under measurement. The stainless steel tube had an outer diameter of 15 mm. To prevent the occurrence of partial discharges at sharp edges due to high electrical field intensity, stainless steel spheres were affixed to each end of the tube. This assembly was then positioned over the designated number of phases and grounded, particularly when the fault under examination was associated with ground potential (refer to Figure 3).

The CCs were positioned on insulators to elevate them to an adequate height, thereby preventing PDs between the lines and the ground. The locations where the CCs were affixed to the insulators underwent inspection using a UVC-sensitive camera. This inspection was aimed at confirming the absence of discharges at the points of contact.

**Figure 4:**
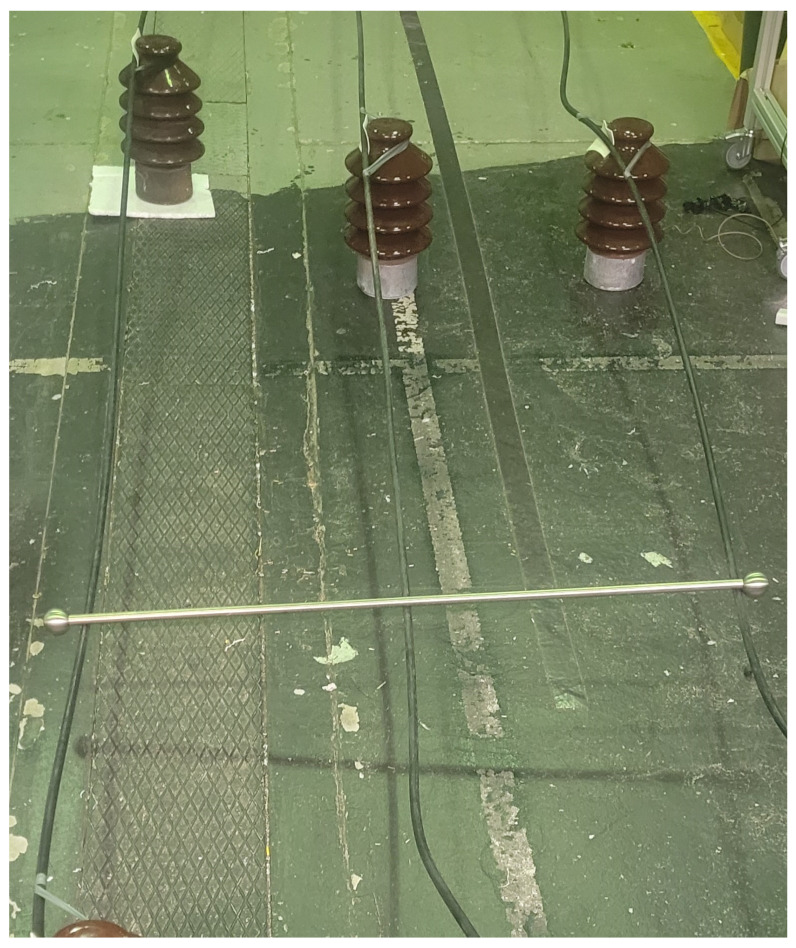
Balanced fault between all three phases.

In Figure 4, the interconnection of all three phases can be observed, interconnected by a stainless steel tube. This configuration demonstrates a scenario with a balanced high-impedance fault occurring between the phases.

### 2.2. Testing on Covered Conductors

In this section, we will outline the process of collecting experimental data. The testing was conducted over a single day to minimize potential external influences, such as weather and time of day. We tested five distinct types of faults, in addition to taking measurements under normal operating conditions (referred to as background measurements). To ensure consistent and reliable results, we adhered to a systematic approach throughout the testing process.

**Figure 5:**
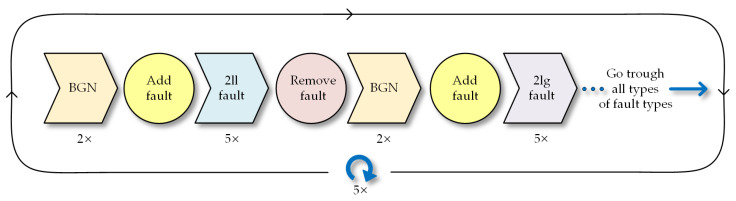
Process of measurements.

For each testing run, we captured the same set of five faults, followed by the change to a different type of fault. Background measurements were taken between these changes in fault types. This approach was chosen to ensure that any incidental background noise or interference could be attributed to a specific type of fault, thereby reducing the possibility of random noise affecting the measurements. The process is depicted in Figure 5.

Throughout the testing procedure, we recorded each sample for a duration of 20 msec, utilizing a sampling frequency of 500 MHz. This high sampling frequency enabled us to capture an entire cycle of the utility frequency, facilitating a precise analysis of the fault conditions.

### 2.3. Dataset Description

The dataset consists of samples from six different classes, comprising five types of faults and one background class. The background class (BGN) comprises 65 samples, while each of the fault classes contains 30 samples. The fault classes are categorized as follows:

- Phase to phase (2 ll);
- Phase to ground (1 lg);
- Phase to phase with ground (2 llg);
- Three phases (3 ll);
- Three phases with ground (3 llg).

**Figure 6:**
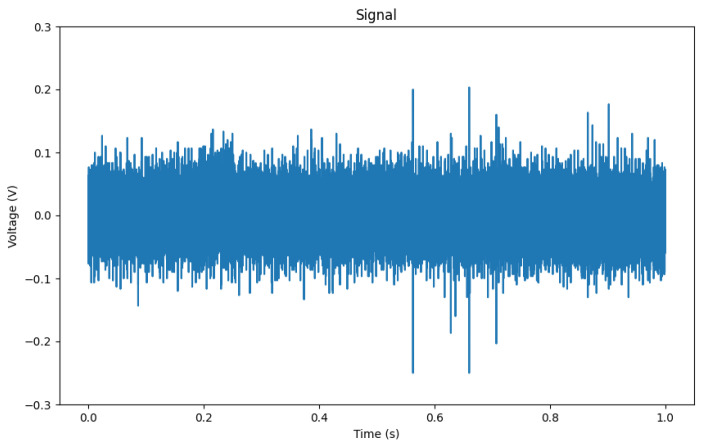
Sample of fault (3llg) with visible PDs.

**Figure 7:**
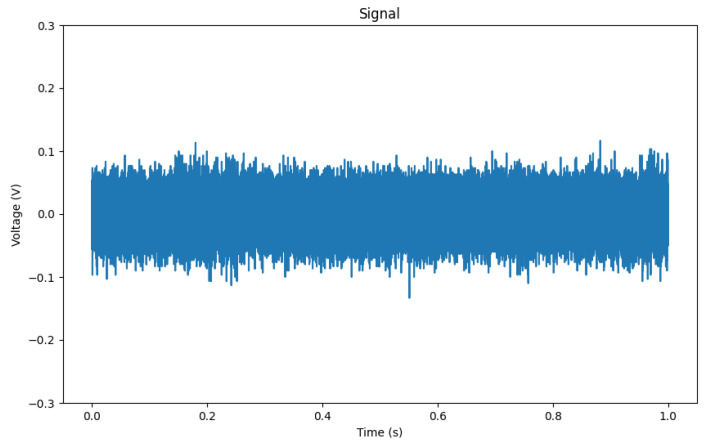
Sample of background (BGN) without any visible PDs.

**Figure 8:**
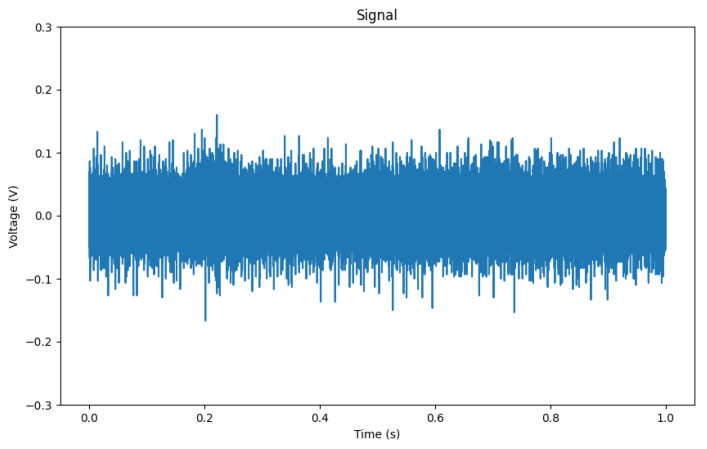
Sample of fault (3llg) without many visible PDs.

Each sample in the dataset comprises 100,000 data points of the floating point data type. These data points are numerical values ranging from −1 to 1, representing the measurements of the electrical system under different fault conditions. The dataset can be partitioned into two main subsets: fault samples (150 samples) and samples without faults (background, 65 samples). Examples of fault patterns can be seen in Figure 6, and examples of background noise in Figure 7. However, PDs are not always that easily visible in the raw signal, as shown in Figure 8. Overview of classes can be seen in Table 1.

Each sample has been stored in the MATLAB file format (.mat), which facilitates convenient access to the data, and allows its manipulation. Each file has a size of 114 MB, making it relatively large to be used directly as raw input for machine learning models.

This dataset is designed for supervised multiclass classification tasks. In a supervised setting, the input data points and corresponding class labels are provided, enabling the training of classification models. Multiclass classification involves assigning a single class label to each input sample from a set of multiple classes. In this case, the classes correspond to different fault types and the background class.

#### 2.3.1. Feature Extraction

Given the substantial size of each sample and the sparsity of the pertinent features, we adopted a two-pronged approach to feature extraction and compared each approach. Our first approach involved extracting two (smaller and extended) diverse sets of statistical features from the signal characteristics (from a single sample). These features ranged from fundamental statistics, such as mean and variance, to more complex spectral measures, like power spectral density (PSD). For a comprehensive list of extracted statistical features, refer to Table A1 in the appendix and the extended set of features in Table A2.

The richness and diversity of these extracted features contribute to providing XGBoost with a substantial amount of information to work with. This, coupled with the algorithm’s inherent ability to handle a large number of features, makes it well suited for scenarios where dimensionality reduction may not be necessary. Additionally, the models are then used in an ensemble, further alleviating the need for dimensionality reduction. Moreover, some works show that reducing or selecting features for XGBoost do not help or even harm the results [28,29].

In addition to statistical features, we employed the MiniRocket algorithm [30], a variant of the ROCKET algorithm [31], which allows us to directly work with raw time-series data. Given the immense size of individual samples (117 MB), we applied max pooling to downsample the signals. The choice of max pooling was motivated by its ability to preserve the distinctive peaks within the data, which are indicative of the fault types. This approach facilitated the effective use of the MiniRocket algorithm, which excels at processing raw time-series data in a very small amount of time while maintaining strong performance.

#### 2.3.2. XGBoost Algorithm

For the classification task, the choice of algorithm was influenced by the need for both robust performance and computational efficiency. The XGBoost algorithm [32], a prominent gradient boosting framework, was selected due to its proven track record of achieving high accuracy in complex classification tasks. Its ensemble-based architecture, which combines the predictive power of multiple decision trees, has been well established for its ability to capture intricate relationships within data.

#### 2.3.3. MiniRocket Algorithm

Furthermore, the MiniRocket algorithm [30] was introduced into our classification pipeline to address the unique challenges posed by the dataset. MiniRocket’s accelerated feature extraction capabilities, coupled with its aptitude for handling large datasets and maintaining classification accuracy, made it an ideal complement to XGBoost. However, given the considerable sample size, a challenge emerged in terms of computational efficiency. To overcome this, we applied max pooling to downsample the signal. Max pooling was selected as the downsampling method to retain the crucial peaks that characterize PD events within the time series.

We employed a classification pipeline in which we used the scikit-learn library [33], using the MiniRocket transformer from the sktime library [34], followed by normalization (StandardScaler). Afterward, we used logistic regression with cross-validation (LogisticRegressionCV), as it demonstrated better performance in our preliminary evaluations compared to the recommended ridge classifier, despite having a slower training time.

Eventually, the choice of XGBoost and Rocket algorithms was driven by the specific characteristics of our dataset. The ability of the XGBoost algorithm to learn complex relationships from extracted statistical features complements the capacity of the Rocket algorithm to process raw time series data efficiently. Together, these approaches enhance our ability to perform accurate fault classification in the presence of sparse yet informative PD features within the large-scale electrical system dataset.

### 2.4. Stacking Ensemble

The fundamental concept underlying stacking involves training a meta-model that learns to blend the outputs of individual base models [35]. This meta-model, often termed the “stacking model”, takes predictions from the base models as input and acquires the ability to assign distinct weights to these predictions based on their performance and characteristics. By enabling the meta-model to ascertain the optimal approach for combining predictions, stacking frequently yields improved predictive performance compared to utilizing each base model independently [36].

This assertion is supported by our prior research, which also employed the ensemble technique with simpler models [37,38]. Furthermore, our preliminary assessments revealed that our two methods accurately identify different samples, thus exhibiting complementary behavior. Through the process of stacking, we aim to amalgamate these models, harnessing their collective intelligence to enhance our overall predictive capacity.

We developed a stacking ensemble comprising five distinct machine learning models with varying performance characteristics. Specifically, one of these models was an XGBoost algorithm implemented on an extended feature set, as referenced in Table 2. Additionally, another XGBoost model was employed, this time utilizing a smaller set of features, as indicated in Table 3.

Furthermore, we trained three MiniRocket models, each on a different window size for max pooling, employing max pooling with varying window sizes (1000, 500, and 250). It is worth noting that the max pooling process was applied to the absolute values of the data, while retaining the original sign within the resulting downsampled samples.

**Figure 9:**
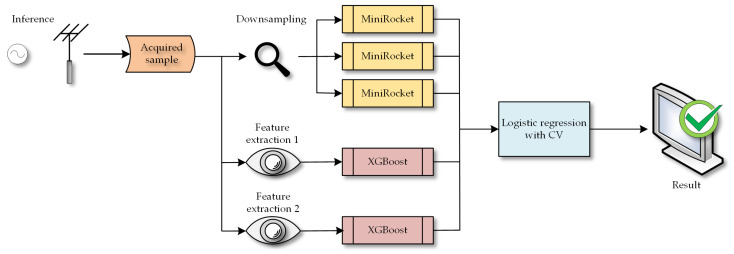
Example of inference and classification of the ensemble.

Subsequently, we employed logistic regression with cross-validation (LogisticRegressionCV) as a meta-learner to fit and combine the individual models. An example of inference and the whole algorithm can be seen in Figure 9.

Combining XGBoost with the ROCKET algorithm creates a potent ensemble method. XGBoost is adept at handling various data types and mitigating overfitting. Meanwhile, ROCKET employs a time-series transformation approach to extract intricate patterns from the data. Merging these capabilities through stacking improves predictive accuracy, stability, and reliability. This ensemble method surpasses individual algorithms by capitalizing on their complementary strengths. Additionally, integrating this method into practical applications is as straightforward as employing any other machine learning algorithm, since it is a fusion of existing algorithms. Standard libraries for these algorithms are available in many languages and can be easily implemented.

### 2.5. Training Process of Proposed Ensemble

The training process was performed on a training set with a size of 0.8% (172 samples), and we stratified the sets to ensure that all classes were represented. Each base model was trained separately on the entire training set. We trained an XGBoost on the extended feature set, an XGBoost on a smaller set of features, and three MiniRocket classifiers were trained on differently downsampled data (with varying window sizes for max pooling).

**Figure 10:**
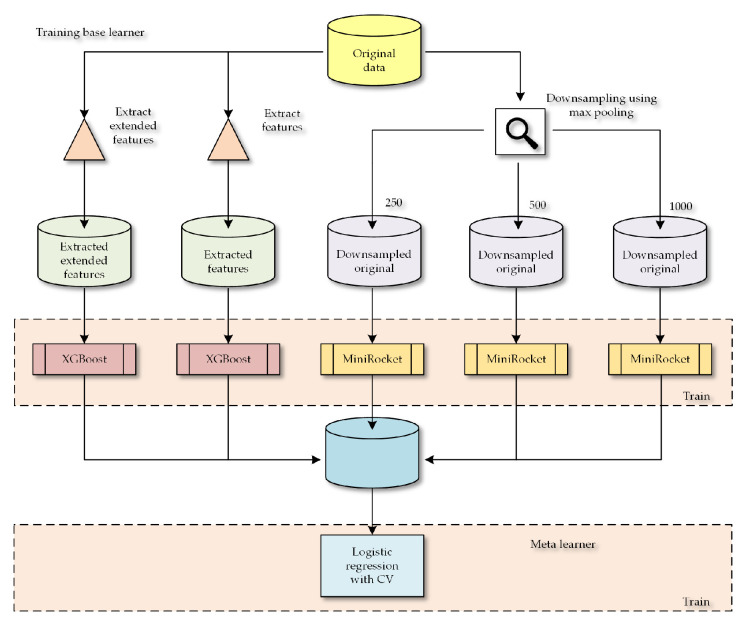
Training process for stacking ensemble.

The output (probabilities of classification from the classifiers) of these five base models were then used as input for the meta-learner, which was a logistic regression with cross-validation. This was also trained on the entire training set. For the XGBoost and MiniRocket algorithms, we used the default settings. The whole process can be seen in Figure 10, where the flow of training inputs is depicted.

## 3. Results and Discussion

In this section, we will present the results of our proposed ensemble, which combines different types of classification and their components. We will engage in a comprehensive discussion of our findings. Next, we will compare our results to those of other algorithms. Finally, we will present the extended results of our proposed ensemble, including training and inference time, window size for downsampling, and the importance of features in the XGBoost algorithm. Our experimentation encompassed the following classification tasks:

1. Fault type classification alongside background assessment.
2. Determination of the presence of PDs within samples.
3. Fault type classification without the inclusion of background samples.
4. Importance of features from the XGBoost algorithm.
5. Comparison between other state-of-the-art algorithms.

### 3.1. Results for Our Proposed Ensemble and Its Parts

In this section, we present the results of our proposed ensemble and its parts and show that the proposed ensemble outperforms its parts and shows high performance.

We employed 100 rounds of random cross-validation, utilizing a test size of 0.2% (resulting in 43 samples for testing), while also ensuring stratified classes. This approach was chosen to avoid overemphasizing any particular fault type.

#### 3.1.1. Evaluation Fault Type Classification alongside Background Assessment

This section presents an in-depth analysis of the performance of our ensemble approach for fault classification. We conducted a comprehensive evaluation of the accuracy of individual ensemble components, followed by an assessment of the overall accuracy achieved by the entire ensemble.

#### 3.1.2. Accuracy Assessment of Ensemble Components

The ensemble encompasses diverse models, including an XGBoost classifier employing a feature-rich set, as well as variants of the MiniRocket model distinguished by differing window sizes. Furthermore, we incorporated an XGBoost model utilizing a reduced feature set, tailored to enhance the efficiency. Table 4 shows the cases of the accuracy scores achieved by these individual ensemble components.

**Figure 11:**
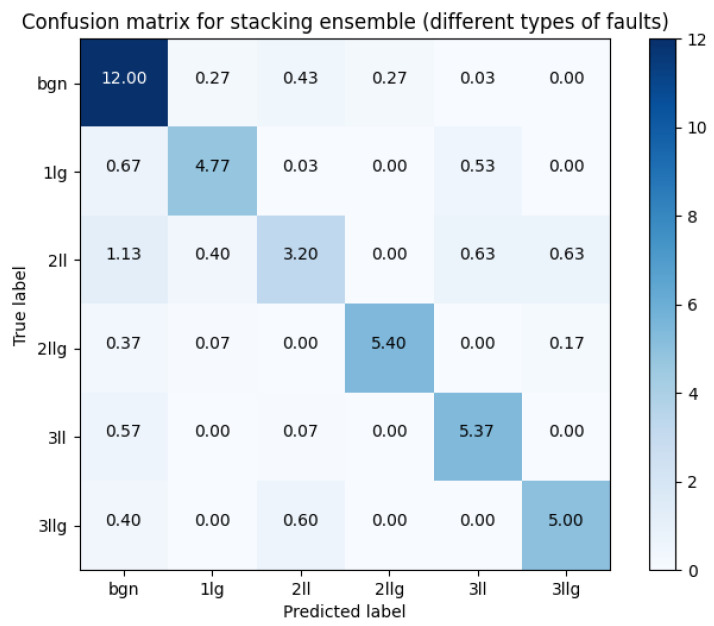
Confusion matrix for stacking ensemble for detecting fault types and background.

The comprehensive ensemble strategy, integrating predictions from diverse components, achieved a total accuracy of 0.8310. This remarkable result underlines the efficacy of our ensemble approach in fault classification tasks. Additionally, we can see a very high precision in the detection of no-fault (BGN) situations, as can be seen in the mean confusion matrix plot presented in Figure 11, in which the precision is nearly 99.8%.

Moreover, as can be seen in Table 4, the minimum and maximum accuracy is best for stacking the ensemble, thus ensuring the best possible results when used instead of a single model. In addition, in Table 5, a comprehensive list of performance metrics for the proposed ensemble can be observed. These metrics include true positives (TP), true negatives (TN), false negatives (FN), accuracy, precision, and recall for the specified class. Notably, the false positive rate for the background class is very low. This is a crucial metric, as a high false positive rate would make the proposed method challenging to use, leading to unnecessary power line inspections. It is also worth noting that faults in real environments occur infrequently. Even though the false positive rate is rather low, the best case would be near zero to have a deployable algorithm. This can be enabled with more data, special techniques, and training methods [39,40], or enabling human validation (human-in-the-loop).

#### 3.1.3. Test of Statistical Significance

For testing statistical significance, we used the Mann–Whitney U test, a non-parametric test designed to ascertain if there are notable disparities between two groups of independent data samples. When extended to multiple sets of data, as is the case here, the test can reveal whether the distributions of scores within these sets exhibit statistically significant variations.

Table A1 presents the statistical significance values between different machine learning methods, with each method being represented by a numeric index. The methods are identified as follows: XGBoost with an extended feature set, MiniRocket with 1000 features, MiniRocket with 500 features, MiniRocket with 250 features, XGBoost with a reduced feature set, and a Stacking ensemble method. The table values denote the *p*-values of statistical significance between the methods. The diagonal values are all 1000, indicating that a method is perfectly correlated with itself.

Table A2 displays a comparison of the methods, but instead of using numerical indexes, the methods are labeled with their respective names. The symbols “-” and ”NS” indicate no statistical significance, while “*”, “**”, and “***” indicate increasing levels of significance (*—*p* < 0.05, **—*p* < 0.01, ***—*p* < 0.001).

In Table A2, the diagonal cells show “-”, indicating that a method is being compared to itself, and hence, there is no meaningful comparison.

These results show that there is no statistical significance in using XGBoost or MiniRocket in terms of accuracy, meaning that they are comparable. On the other hand, the stacking ensemble is more statistically significant in terms of accuracy than the others.

#### 3.1.4. Differences between Fault and Non-Fault Instances

**Figure 12:**
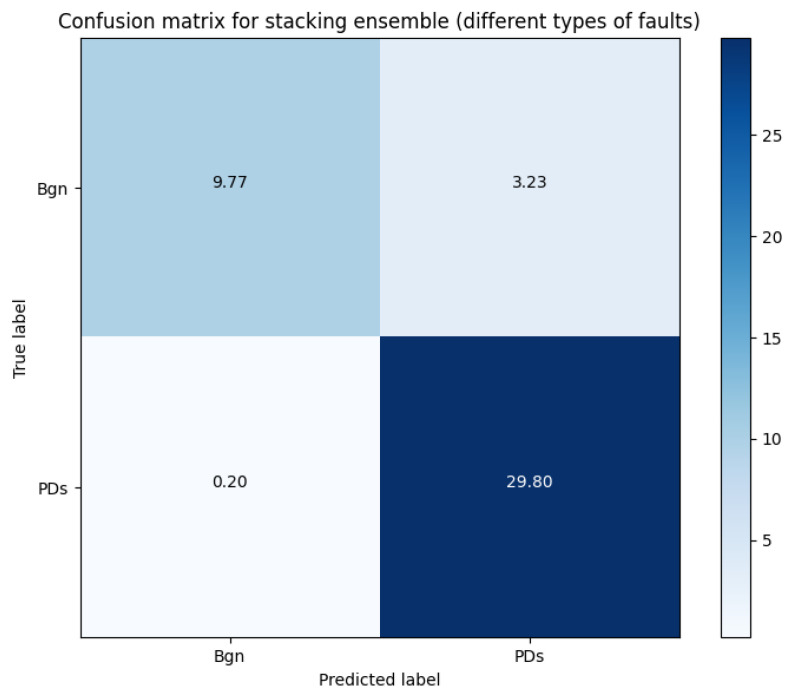
Confusion matrix for stacking ensemble for detecting fault (PDs) or without fault.

Table 6 presents the accuracy of ensemble components in distinguishing between fault and non-fault samples. This table displays the accuracy levels attained by individual ensemble components when tasked with discerning between instances with faults and those without faults. The predictive accuracy of each ensemble component is assessed, serving as an indicator of its effectiveness in addressing the underlying classification challenge. The confusion matrix with TP, FP, TN, and FN can be seen in Figure 12.

The best-performing ensemble component was once again the stacking ensemble. Interestingly, the disparity in performance between the parts-based and whole-stacking ensembles was not significantly pronounced, with the best-performing ensemble outperforming the others by only 2%. These results indicate that our proposed method is proficient in detecting PDs or determining their absence.

Interestingly, the precision of detection is slightly lower (98%) when compared to the classification of types (99.8%) for all types of detection. This suggests that classifying different classes of faults could potentially aid in the overall detection of faults.

We theorize that relying solely on identifying faults or non-fault instances may increase the false positive rate. Additionally, utilizing fault types may improve the precision of this process. It is also possible that an unbalanced dataset, with more samples containing faults compared to fewer samples without faults, could be a contributing factor to why this experiment performed less favorably in these aspects compared to the classification based on fault types.

#### 3.1.5. Differences between Types of Fault Only

**Figure 13:**
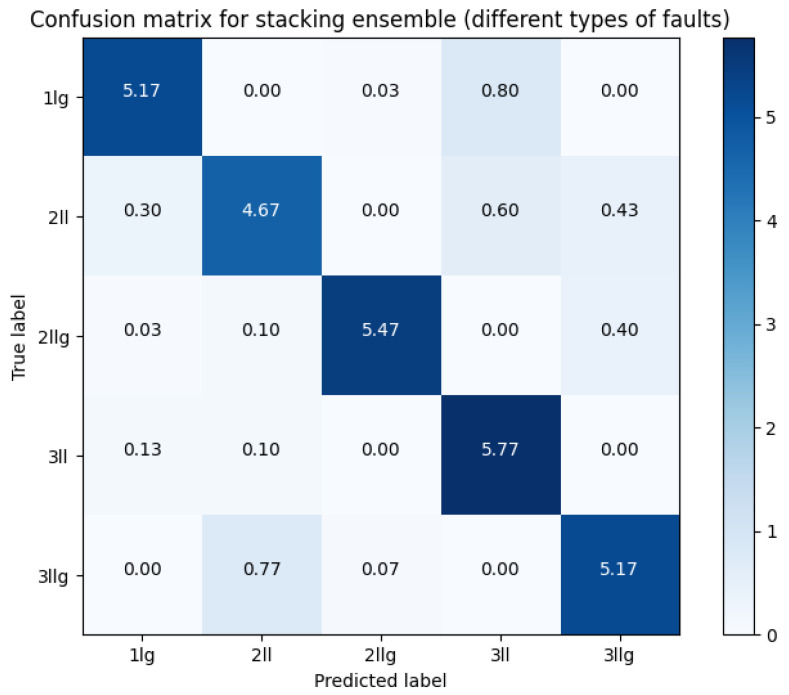
Confusion matrix for stacking ensemble for detecting fault types only.

We attempted to exclusively distinguish between various types of faults. This effort was aimed at revealing both the similarities among different fault types and cases where classes could be misidentified. Figure 13 illustrates that there are relatively few cases of misclassification. Among these, the most commonly misclassified classes are 3 ll, which is occasionally mistaken for 2 ll, and 1 lg. Interestingly, there does not appear to be a clear pattern explaining why certain types are misclassified as others. However, it is worth noting that there are no cases of misclassification between grounded and non-grounded connections when the number of phases is the same (e.g., 2 ll vs. 2 llg or 3 ll vs. 3 llg).

We found that there are no classes similar enough to pose a challenge in distinguishing them. While we initially speculated about this possibility, it is intriguing to note that each type of fault exhibits distinct characteristics. This underscores the feasibility of fault-type classification and emphasizes the need for further research to enhance the reliability and utilization of covered conductors in distribution power lines.

The most accurate variant can be observed in Table 7, which is the stacking ensemble. Interestingly, MiniRocket with a window size of 250 comes in as the second best, which is intriguing considering its underperformance in previous results. This observation might indicate that the components of our ensemble are quite diverse. This diversity could be the reason behind the overall improvement in the classification results.

### 3.2. Comparison to Other Algorithms

The classification results are presented in Table 8. Various classifiers, including deep learning models like TapNet, InceptionTime, and LSTMFCN, as well as traditional methods, such as logistic regression and support vector machine, were employed using different libraries. Notably, our proposed ensemble of Rocket and XGBoost algorithms demonstrated superior performance, outperforming all other classifiers with an accuracy of 0.842. It is worth mentioning that TapNet, a deep learning model, could not be evaluated on an NVIDIA T4 GPU due to its memory limitations.

This shows that a combination of two different but successful algorithms can improve performance. Interestingly, deep learning, which is supposed to be applied for time series classification, performed very poorly and required large hardware resources. It is probable that increasing the training dataset would improve these results, but the very large memory consumption would largely limit these algorithms.

Our results indicate that our proposed model outperforms other algorithms. Additionally, utilizing heterogeneous models as base learners with distinct input features (downsampled raw input and extracted features) yields superior results compared to other algorithms.

### 3.3. Analysis of Proposed Ensemble for Detecting Type of Fault

In this section, we present other interesting results for our proposed ensemble.

#### 3.3.1. Training and Inference Time

Table 9 provides insights into the inference and average training times for various classifiers. Notably, the ensemble classifier exhibits a relatively large average training time, primarily due to training five different classifiers as part of the ensemble strategy. However, its inference time remained quite acceptable and competitive. These results were acquired on an Intel(R) Xeon(R) CPU E5-2630, where 30 trials were run for each benchmark.

Interestingly, algorithms used for time series classification, such as WEASEL and MiniRocket, tend to be slow in terms of training time. It is important to note that our MiniRocket algorithm did not use the Ridge classifier, as was originally proposed [31], but instead, it employed linear regression with cross-validation. Although this approach is slower, it yields superior results.

If the speed is of greater importance than the accuracy, XGBoost stands out as an excellent choice with minimal training and near-instantaneous inference times. It offers a balance between the training speed and the inference efficiency, making it a preferred option for real-time applications where rapid predictions are crucial. Additionally, it should be noted that the ensemble form is not the best possible one yet and there are many potential improvements, like bagging, using subsets of the dataset, and pruning the ensemble. These methods could greatly enhance the training process.

#### 3.3.2. Influence of Window Size for Rocket Algorithm

**Figure 14:**
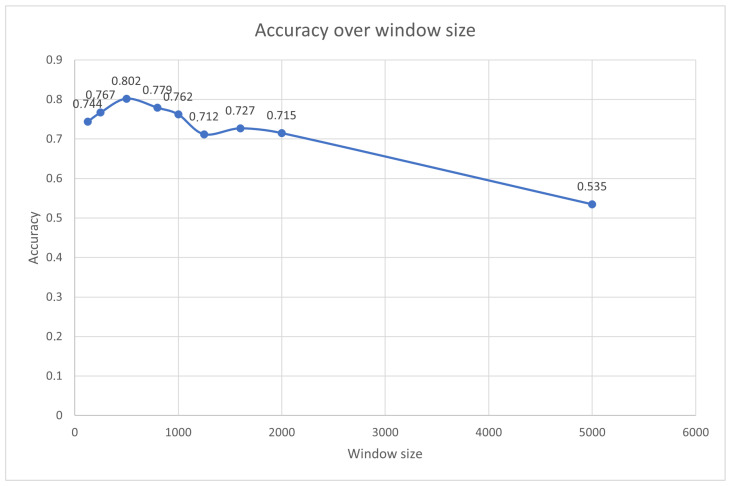
Accuracy of MiniRocket algorithm over the window size of the downsampled signal.

Table 10 presents the performance of the Rocket algorithm with various window sizes for max pooling the data. It is evident that the accuracy tends to fluctuate with changes in the window size, as can be seen in Figure 14. Notably, smaller window sizes, such as 125 and 250, yield a higher accuracy, suggesting that they effectively highlight finer-grained features relevant for classifying partial discharges.

On the other hand, larger window sizes, like 5000, correspond to smaller input sizes and result in a lower accuracy, indicating that they may compress the signal too much, potentially losing critical information related to partial discharges.

Interestingly, a window size of 500 emerges as the most effective choice, indicating that it strikes a balance between capturing important peak features and avoiding over-compression, leading to the highest average accuracy in this particular evaluation.

#### 3.3.3. Importance of Features in XGboost Component of the Ensemble

We conducted an investigation into the importance of features within the XGBoost algorithm, leveraging its inherent interpretability. The analysis of feature importance serves a dual purpose: it enhances comprehension of the factors that steer predictions and provides avenues for refining models and exploring data. By pinpointing the most influential features, practitioners can make informed decisions regarding feature selection, engineering, and potential model enhancements.

In Table 11 and Table 12, we present the top 10 features recognized by the XGBoost algorithm as exerting the greatest influence on prediction outcomes within the context of a reduced feature set. The associated values for each feature denote their respective importance scores, indicating their contributions to the decision-making process of the model. A higher score signifies a more pronounced impact on predictions.

The data presented in Table 11 illustrate that the most crucial features within the reduced set are closely associated with peaks and their variations. This observation aligns with the fact that the signal representation of PDs is manifested as peaks in the time–amplitude domain.

The results presented in Table 12 highlight the significance of various features derived from the extended set. Among these features, the crest factor stands out as the most important element, with an importance value of 0.126. The crest factor, as defined here, captures the relationship between the peak amplitude and the RMS value of the waveform. Its high importance value suggests that this metric plays a crucial role in identifying cases of prominent peaks in the waveform. Since higher crest factors indicate more pronounced peaks, their relevance to the detection of PDs and their types aligns with expectations.

Continuing down the list, the subsequent features exhibit descending importance values. The mean peak prominence 2, peak-to-peak value, number of peaks 2, and peak height range all contribute significantly to the overall assessment of the waveform. These characteristics inherently encapsulate information about the peaks and variations present, further aiding in the identification of potential PDs. Additionally, the inclusion of features from the spectral domain, such as maximum frequency and spectral centroid, indicates the value of frequency-based information in this context. These spectral features, while not as dominant as the crest factor, still offer meaningful insights that enhance the overall analysis.

The importance of all features can be observed in Table A3 for the reduced set of features and in Table 12 for the extended set of features. Features that do not influence the results can be observed in these tables and can be subsequently removed from the training process. We chose to retain them in order to demonstrate that the XGBoost algorithm is capable of learning significant features. This also contributes to the explainability of the feature set.

## 4. Conclusions

In this study, we present a novel method for detecting fault types in covered conductors used in medium-power transmission lines. We simulated five possible fault types along with the presence of noise. Our proposed stacking ensemble approach achieved a mean accuracy of 0.84%, demonstrating high precision in fault detection.

### 4.1. Remarks

We have demonstrated that our proposed method can effectively distinguish between different types of faults while maintaining a low false positive rate. This is crucial for real-world deployment. It signifies that fault detection using antennas is not only feasible but can also be further developed for practical implementation after additional research.

We employed a heterogeneous stacking ensemble method, which we believe is innovative in the field of partial discharges. This method utilizes two different types of inputs. Our proposed ensemble outperformed both state-of-the-art and classical machine learning algorithms. Additionally, we investigated the optimal window size for downsampling. We showed that using an ensemble had statistical significance instead of using the components of an ensemble alone.

Furthermore, we identified crucial features that are utilized by the XGBoost algorithm, adding a layer of explainability to our approach. While the training time is relatively high due to the training of six different classifiers, the inference time is comparable to other methods, indicating its high usability.

### 4.2. Limitations and Future Work

The limitations of our study were primarily linked to the size of the acquired dataset. Despite this constraint, we maintain that the amount of data was adequate for the scope of this work. In future research, a more substantial dataset would be preferable. Additionally, an intriguing avenue for exploration would be to broaden the components of the stacking ensemble or investigate alternative classification methods, including more complex deep learning models or diverse stacking schemes. Moreover, employing feature engineering could offer valuable insights and would be an interesting aspect to consider. A limitation regarding implementation in real environments would most certainly be the selection of hardware components that would have the required accuracy and computing power while being cost-effective and having low consumption. Another issue could be caused by the power source for the measuring station, since the aim is to avoid the usage of high-voltage transformers which would require the shutdown of the power line. The solution for this could be the usage of a photovoltaic panel in combination with a battery. There is also a requirement for GSM network availability, since the information has to be sent to a distribution network operator.

### 4.3. Summary

In conclusion, this paper introduces a comprehensive and innovative approach to enhance the detection of fault types, which was previously carried out only as fault detection, in covered conductors within distribution power lines. This study employs an antenna-based method to detect faults and a unique fault-type classification technique utilizing a stacking ensemble that combines XGBoost and ROCKET algorithms. This shows a possible way to address the demand for accurate fault type detection in XLPE-covered conductors. The proposed classifiers exhibit remarkable accuracy in distinguishing between different fault types. The integration of the heterogeneous stacking ensemble method further bolsters accuracy, potentially leading to the wider adoption of covered conductors. This advancement has the capacity to reduce buffer zones, enhance power delivery reliability, and contribute to positive environmental outcomes by preventing accidents and promoting the secure and extensive utilization of covered conductors in distribution power lines.

## Figures and Tables

**Table 1:** Dataset summary.

Class	Number of Samples
Background (BGN)	65
Phase to Phase (2ll)	30
Phase to Ground (1lg)	30
Phase to Phase with Ground (2llg)	30
Three Phases (3ll)	30
Last Three Phases with Ground (3llg)	30
Total	215

**Table 2:** Extended extracted features.

Feature	Description/Parameters
(Features from Table 3)	
Frequency Domain Features	
Max Frequency	Frequency corresponding to the maximum power in power spectrum
Mean Power	Mean power in the power spectrum
Standard Deviation Power	Standard deviation of power in the power spectrum
Peak-to-Peak	Difference between maximum and minimum data values
Interquartile Range	Interquartile range of the data
Spectral Centroid	Weighted average of frequencies in the power spectrum
Spectral Spread	Spread of frequencies in the power spectrum
Spectral Skewness	Skewness of the power spectrum
Spectral Kurtosis	Kurtosis of the power spectrum
Spectral Entropy	Entropy of the power spectrum
Temporal Features	
Zero Crossing Rate	Number of times signal crosses zero
RMS Energy	Root mean square energy of the data
Crest Factor	Ratio of peak value to RMS energy
Form Factor	Ratio of RMS energy to mean absolute value
Spectral Roll-off	Frequency below which a certain percentage of the total power lies
Harmonic-to-Noise Ratio (HNR)	Ratio of harmonic energy to noise energy
Fundamental Frequency	Frequency with maximum power in power spectrum
Ratio of Harmonic Energies	Ratio of harmonic energy to total energy
Peak-to-Average Ratio (PAR)	Ratio of peak value to mean absolute value
Dominant Frequency	Frequency with maximum power in power spectrum
Mean Frequency	Weighted average frequency in the power spectrum
Signal Entropy	Entropy of the power spectrum
Normalized L2 Norm	L2 norm normalized by data length
Signal-to-Noise Ratio (SNR)	Ratio of mean to standard deviation

**Table 3:** Extracted features.

Feature	Description/Parameters
Peak Features	Adjusted peak detection:
	Distance = 5000
Mean Peak Prominence	Mean of the peak prominences of detected peaks
Num Peaks	Number of detected peaks
Mean Peak Height	Mean height of detected peaks
Standard Deviation Peak Height	Standard deviation of peak heights
Peak Height Range	Range between the maximum and minimum peak heights
Peak Height Ratio	Ratio of maximum peak height to minimum peak height
Peak Features	Adjusted peak detection:
	Distance = 50,000
Mean Peak Prominence 2	Mean of the peak prominences of detected peaks (adjusted)
Num Peaks 2	Number of detected peaks (adjusted)
Mean Peak Height 2	Mean height of detected peaks (adjusted)
Standard Deviation Peak Height 2	Standard deviation of peak heights (adjusted)
Peak Height Range 2	Range between the maximum and minimum peak heights (adjusted)
Peak Height Ratio 2	Ratio of maximum peak height to minimum peak height (adjusted)
Statistical Features	
Skewness	Skewness of the data distribution
Kurtosis	Kurtosis of the data distribution
Variance	Variance of the data
Mean	Mean of the data
Standard Deviation	Standard deviation of the data
Median	Median of the data
Maximum	Maximum value in the data
Minimum	Minimum value in the data
Root Mean Square (RMS)	Square root of the mean of squared data values
Sum of PSD	Sum of power spectral density using Welch’s method

**Table 4:** Accuracy of ensemble components in distinguishing all captured types.

Ensemble Component	Accuracy	Min Accuracy	Max Accuracy
XGBoost (All Features)	0.78	0.69	0.88
MiniRocket (Window Size 1000)	0.74	0.62	0.84
MiniRocket (Window Size 500)	0.79	0.65	0.91
MiniRocket (Window Size 250)	0.77	0.65	0.88
XGBoost (Reduced Features)	0.72	0.63	0.83
Stacking Ensemble	0.84	0.72	0.98

**Table 5:** Performance of ensemble in distinguishing all captured types.

Type	TP	FP	FN	TN	Total	Acc.	Prec.	Recall
bgn	12.0	3.1	1.0	26.9	43.0	0.9	0.8	0.9
1lg	4.8	0.7	1.2	36.3	43.0	1.0	0.9	0.8
2ll	3.2	1.1	2.8	35.9	43.0	0.9	0.7	0.5
2llg	5.4	0.3	0.6	36.7	43.0	1.0	1.0	0.9
3ll	5.4	1.2	0.6	35.8	43.0	1.0	0.8	0.9
3llg	5.0	0.8	1.0	36.2	43.0	1.0	0.9	0.8

**Table 6:** Accuracy of ensemble components between fault and non-fault types.

Ensemble Component	Accuracy
XGBoost (All Features)	0.90
MiniRocket (Window Size 1000)	0.86
MiniRocket (Window Size 500)	0.89
MiniRocket (Window Size 250)	0.89
XGBoost (Reduced Features)	0.90
Stacking Ensemble	0.92

**Table 7:** Accuracy of ensemble components between types of fault.

Ensemble Component	Accuracy
XGBoost (All Features)	0.78
MiniRocket (Window Size 1000)	0.83
MiniRocket (Window Size 500)	0.84
MiniRocket (Window Size 250)	0.85
XGBoost (Reduced Features)	0.74
Stacking Ensemble	**0.88**

**Table 8:** Classification results with different classifiers (* TapNet was unable to fit a memory of NVIDIA T4 GPU).

Classifier	Type	Library	Accuracy
TapNet	Deep Learning	Sktime	N/A *
InceptionTime	Deep Learning	Sktime	0.140
Logistic Regression with CV	Traditional	Scipy	0.248
LSTMFCN	Deep Learning	Sktime	0.349
Support Vector Machine	Traditional	Scipy	0.302
Multi-Layer Perceptron	Neural network	Scipy	0.302
Convolutional Neural Network	Deep Learning	Sktime	0.302
Time Series Forest	Traditional Time Series	Sktime	0.370
WEASEL	Dictionary Based	Sktime	0.533
Catch22	Feature-based	Sktime	0.634
Random Forest	Traditional	Scipy	0.601
Supervised Time Series Forest	Interval Based	Sktime	0.772
XGBoost	Traditional	Xgboost	0.781
Rocket	Kernel Based	Sktime	0.785
Ensemble	Custom	-	0.842

**Table 9:** Inference and average training times for different classifiers.

Classifier	Avg Train Time (s)	Inference Time (s)
XGBoost	0.19	0.00
Logistic Regression with CV	0.39	0.00
Support Vector Machine	0.00	0.00
Multi-Layer Perceptron	0.05	0.00
Random Forest	0.43	0.10
Convolutional Neural Network	5.72	0.16
Time Series Forest	3.45	0.86
MiniRocket	204.63	0.45
InceptionTime	209.63	1.08
Ensemble	640.45	1.38
LSTMFCN	86.80	1.51
Supervised Time Series Forest	5.69	2.01
WEASEL	120.48	3.76
Catch22	38.85	9.70

**Table 10:** Performance of Rocket algorithm with different window sizes for max pooling the data. The values represent the average accuracy over 30 random cross-validations.

Window Size	Size of Sample	Accuracy
125	800,000	0.744
250	400,000	0.767
500	200,000	0.802
800	125,000	0.779
1000	100,000	0.762
1250	80,000	0.712
1600	62,500	0.727
2000	50,000	0.715
5000	20,000	0.535

**Table 11:** Top 10 features for the reduced feature set.

Feature	Importance Value
Peak Height Range	0.136
Maximum	0.111
Mean Peak Prominence 2	0.098
Number of Peaks 2	0.083
Standard Deviation Peak Height 2	0.078
Standard Deviation Peak Height	0.070
Mean Peak Height 2	0.069
Minimum	0.048
Peak Height Ratio 2	0.043
Peak Height Ratio	0.039

**Table 12:** Importance values of extended set features.

Feature	Importance Value
Crest Factor	0.126
Mean Peak Prominence 2	0.065
Peak-to-Peak	0.061
Number of Peaks 2	0.057
Peak Height Range	0.057
Maximum Frequency	0.055
Standard Deviation Peak Height	0.046
Variance	0.046
Maximum Value	0.042
Spectral Centroid	0.041

## Data Availability

Data sharing is not applicable.

## Abbreviations

The following abbreviations are used in this manuscript:

CC	Covered conductors
PD	Partial discharges
SNR	Signal-to-noise ratio
HNR	Harmonic-to-noise ratio
PSD	Power spectral density
UVC	Short-wave UV
XLPE	Cross-linked polyethylene
BGN	Samples containing background
2ll	Phase to phase
1lg	Phase to ground
2llg	Phase to phase with ground
3ll	Three phases
3llg	Three phases with ground
TP	True positive
FP	False positive
TN	True negative
FN	False negative

## Appendix A

**Table A1:** Statistical significance: *p*-values of fault type and background classification.

	XGBoost	MiniRocket 1000	MiniRocket 500	MiniRocket 250	XGBoost Reduced	Stacking
XGBoost	1.000000	$1.106405\times 10^{-3}$	0.193134	0.796693	$7.765196\times 10^{-6}$	$1.081966\times 10^{-5}$
MiniRocket 1000	0.001106	1.000000	0.000047	0.001811	$1.113019\times 10^{-1}$	$1.902296\times 10^{-11}$
MiniRocket 500	0.193134	$4.681914\times 10^{-5}$	1.000000	0.103009	$1.413209\times 10^{-6}$	$3.760277\times 10^{-3}$
MiniRocket 250	0.796693	$1.810803\times 10^{-3}$	0.103009	1.000000	$1.853162\times 10^{-5}$	$1.221413\times 10^{-6}$
XGBoost Reduced	$7.765196\times 10^{-6}$	$1.113019\times 10^{-1}$	$1.000000\times 10^{-10}$	$1.900000\times 10^{-5}$	1.000000	$2.785192\times 10^{-12}$
Stacking	$1.081966\times 10^{-5}$	$1.902296\times 10^{-11}$	$3.760277\times 10^{-3}$	$1.221413\times 10^{-6}$	$2.785192\times 10^{-12}$	1.000000

**Table A2:** Sign plot for statistical significance of fault type and background classification.

	XGBoost	MiniRocket 1000	MiniRocket 500	MiniRocket 250	XGBoost Reduced	Stacking
XGBoost	-	**	NS	NS	***	***
MiniRocket 1000	**	-	***	**	NS	***
MiniRocket 500	NS	***	-	NS	***	**
MiniRocket 250	NS	**	NS	-	**	***
XGBoost Reduced	***	NS	***	**	-	***
Stacking	***	***	**	***	***	-

**Table A3:** Importance of extracted features for XGboost model with reduced features set.

Feature	Importance
Peak Features	Adjusted peak detection:
	Distance = 5000
Mean Peak Prominence	0.097632
Num Peaks	0.031346
Mean Peak Height	0.015803
Standard Deviation Peak Height	0.069656
Peak Height Range	0.136260
Peak Height Ratio	0.039222
Peak Features	Adjusted peak detection:
	Distance = 50,000
Mean Peak Prominence 2	0.097632
Num Peaks 2	0.083282
Mean Peak Height 2	0.068708
Standard Deviation Peak Height 2	0.078199
Peak Height Range 2	0.004834
Peak Height Ratio 2	0.042518
Statistical Features	
Skewness	0.027511
Kurtosis	0.027172
Variance	0.029464
Mean	0.020554
Standard Deviation	0.000000
Median	0.000000
Maximum	0.111720
Minimum	0.047870
Root Mean Square (RMS)	0.004374
Sum of PSD	0.027478

**Table A4:** Importance of extracted features for XGboost model with extended feature set.

Feature	Importance
Peak Features	Adjusted peak detection:
	Distance = 5000
Mean Peak Prominence	0.018827
Number of Peaks	0.016362
Mean Peak Height	0.011084
Standard Deviation of Peak Height	0.045729
Peak Height Range	0.056671
Peak Height Ratio	0.015543
Peak Features	Adjusted peak detection:
	Distance = 50,000
Mean Peak Prominence 2	0.065121
Number of Peaks 2	0.057345
Mean Peak Height 2	0.040397
Standard Deviation of Peak Height 2	0.038609
Peak Height Range 2	0.000707
Peak Height Ratio 2	0.023249
Statistical Features	
Skewness	0.010877
Kurtosis	0.011849
Variance	0.045627
Mean	0.010598
Standard Deviation	0.000000
Median	0.000000
Maximum	0.041645
Minimum	0.016252
Variance	0.000000
RMS (Root Mean Square)	0.020945
Spectral and Additional Features	
Sum of Power Spectral Density	0.011494
Maximum Frequency	0.055299
Power Mean	0.000000
Power Standard Deviation	0.022223
Peak-to-Peak	0.061269
Interquartile Range	0.001098
Spectral Centroid	0.041281
Spectral Spread	0.012221
Spectral Skewness	0.023546
Spectral Kurtosis	0.002866
Spectral Centroid	0.000000
Spectral Entropy	0.012583
Zero-Crossing Rate (ZCR)	0.010718
RMS Energy	0.000000
Crest Factor	0.126061
Form Factor	0.021400
Spectral Roll-Off	0.011754
Harmonics-to-Noise Ratio (HNR)	0.017958
Fundamental Frequency	0.000000
Ratio of Harmonic Energy	0.011589
PAR (Peak-to-Average Ratio)	0.001609
Dominant Frequency	0.000000
Mean Frequency	0.000000
Signal Entropy	0.000000
Normalized L2 Norm	0.000000
SNR (Signal-to-Noise Ratio)	0.007596
